# The solvent and treatment regimen of sodium selenite cause its effects to vary on the radiation response of human bronchial cells from tumour and normal tissues

**DOI:** 10.1007/s12032-020-01437-y

**Published:** 2020-11-18

**Authors:** Katrin Manda, Stephan Kriesen, Guido Hildebrandt

**Affiliations:** Department of Radiotherapy and Radiation Oncology, University Medical Center Rostock, Suedring 75, 18059 Rostock, Germany

**Keywords:** Sodium selenite, Ionizing irradiation, A549, BEAS-2B, Cell cycle, Metabolic activity

## Abstract

Sodium selenite is often given to moderate the side effects of cancer therapy to enhance the cellular defence of non-cancerous cells. To determine whether sodium selenite during radiotherapy protects not only normal cells but also cancer cells, which would imply a reduction of the desired effect of irradiation on tumour during radiotherapy, the effect of the combined treatment of irradiation and sodium selenite was investigated. Human bronchial cells from carcinoma (A549) and normal tissue (BEAS-2B) were treated with sodium selenite and effects on growth and in combination with radiation on metabolic activity and cell cycle distribution were studied. The influence on radiosensitivity was determined via colony forming assays using different solvents of sodium selenite and treatment schedules. It was shown that sodium selenite inhibits growth and influences cell cycle distribution of both normal and tumour cells. Metabolic activity of normal cells decreased more rapidly compared to that of cancer cells. The influence of sodium selenite on radiation response depended on the different treatment schedules and was strongly affected by the solvent of the agent. It could be shown that the effect of sodium selenite on radiation response is strongly dependent on the respective experimental in vitro conditions and ranges from lead to an initially suspected but ultimately no real radioprotection to radiosensitizing up to no effect in one and the same cell line. This might be a reason for controversially described cell responses to radiation under the influence of sodium selenite in studies so far.

**Electronic supplementary material:**

The online version of this article (10.1007/s12032-020-01437-y) contains supplementary material, which is available to authorized users.

## Introduction

Selenium as an essential trace element is used as the inorganic form sodium selenite to moderate the side effects of cancer therapy [1] and enhance the cellular defence of healthy cells [2, 3]. The mode of action of sodium selenite is not yet known in detail. The effect appears to be based on different mechanisms. On the one hand, selenite has immunomodulatory functions and was described as positively influencing the immune system. Tumour cells have free sulfhydryl groups on their cell membranes, which protect them from attacks of proteolytic enzymes of phagocytic cells and mediate their uncontrolled growth. Selenite is able to oxidize these free and protein-bound sulfhydryl groups to corresponding disulfides, which inhibits the protective (parafibrin-) barrier of cancer cell membranes and make them vulnerable to the destructive activity of phagocytes [4, 5]. In addition, selenite causes an increase of immunocompetent cells like macrophages and can direct activate natural killer (NK) cells [4, 6].

Selenite—with its unique redox chemistry—shows antioxidant and prooxidant properties. Its concentration, the intracellular redox status as well as the activity of redox-sensitive proteins and enzymes participate whether antioxidant or prooxidant activities prevail. The metabolic pathway of selenite, its redox-active properties in mammalian cells and tissue and its consequences were described in a very detailed manner by Weekley and Harris [7].

Apart from the immunomodulatory effect, it was assumed for a long time that the positive effect of selenite is only caused by its antioxidant properties, which support normal cells to reduce their oxidative stress level. It was, therefore, considered that sodium selenite should be used as a radiation protection agent in normal tissue for the prophylaxis of radiation effects [8, 9]. In studies, it was described that sodium selenite has a radioprotective effect on parotid gland tissue in rats [10]. By lowering the amount of lipid peroxide and increasing glutathione and glutathione peroxidase activity, sodium selenite significantly improved the oxidative stress response of the uterus and ovaries induced by radiation [11]. During whole-body irradiation treatment with sodium selenite, mice were protected against radiation-induced genotoxicity and DNA damage in peripheral leukocytes, but it did not keep the animals from mortality or gastrointestinal and hematopoietic lesions [12]. However, overall, in the further literature, the effects of sodium selenite described on the cellular radiation sensitivity are contradictory. There are reports for sodium selenite from radiosensitizing [13, 14] to radioprotection [15]. Furthermore, in several studies no influence of sodium selenite on radiation response was observed [16, 17].

Meanwhile, toxicity of selenite on tumour cells is described as also being mediated because of its prooxidative character [18]. Selenite is involved in the production of reactive oxygen species (ROS), which leads the tumour cells, among others, to DNA damage, mainly DNA double-stranded breaks, induction of apoptosis, and finally to suppression of cancer progression [19, 20]. Cancer cells are characterized by an altered redox status with increased ROS levels. Therefore, these are likely to be more susceptible to damage from additional oxidative stress attacks caused by drugs [21]. Normal cells, on the other hand, are able to tolerate a certain level of additional exogenous oxidative stress.

The discussion about the effect of the additional treatment with sodium selenite accompanying radiation therapy is still controversial. The main question for our study was whether the radiation-induced effect on cancer cells is disabled by sodium selenite treatment and whether normal cells are protected by this combined treatment. Therefore, in the present study, we evaluated the effect of sodium selenite in combination with ionizing irradiation in vitro. We tested whether treatment with sodium selenite affects radiation response of human bronchial epithelial cells and if there are differences between cells from carcinoma (A549) and normal tissue (BEAS-2B). These cells were chosen as a biological model because lung cancer is one of the most common and serious types of cancer worldwide. In addition, among the various human cancer cell lines that were investigated, cells from lungs seem to be extra sensitive to sodium selenite [22, 23].

## Materials and methods

### Materials

Dulbecco’s Modified Eagle’s medium (DMEM), phosphate buffered saline (PBS) and fetal bovine serum (FBS) were purchased from PAA Laboratories GmbH (Cölbe, Germany). Penicillin/streptomycin (100 U/ml/100 µg/ml) and Trypsin/EDTA were obtained from Biochrom AG, Berlin, Germany. Sodium selenite (Na_2_SeO_3_, Sigma-Aldrich Fine Chemicals, Taufkirchen, Germany) was generally dissolved in physiological sodium chloride solution or in aqua bidest for some of the clonogenic assays (stock solution 1.72 mg/ml). For experiments sodium selenite stock solution was diluted with DMEM to produce the final concentrations.

### Cell lines

Human bronchial carcinoma cells A549 (DSMZ, Braunschweig, Germany; DSMZ no.: ACC 107) and the bronchial epithelial cell line BEAS-2B (European Collection of Cell Cultures, ECACC, Salisbury, UK; Catalogue No.: 95102433) were cultivated at 37 °C, 5% CO_2_ in DMEM, supplemented with 10% FBS and 1% penicillin/streptomycin. The cell lines were passaged once weekly to ensure exponential growth.

### Irradiation

Irradiation was administered at room temperature using a linear accelerator ONCOR Expression (Siemens, Erlangen, Germany) at 3.75 Gy/min (energy 6 MeV) as described before [24]. The irradiation doses used were 0 Gy, 2 Gy, 4 Gy, 6 Gy, and 8 Gy.

### Growth curves

Cells were seeded in a 24-well plate in triplicates for each experimental approach. Twenty four hours after seeding and cell attachment sodium selenite was added once at concentrations of 0 µM to 100 µM and incubated with sodium selenite for 5 days without medium exchange. In a different experimental pattern daily addition of sodium selenite without medium exchange after a 24 h cell adhesion period was performed. Growth curves were created on the mean of three independent experiments.

### Clonogenic assay

Cells were seeded in 75 cm^2^-flasks in an appropriate cell density and treated with the agent (10 µM or 50 µM sodium selenite) 0.5 h or 24 h before irradiation (0 Gy or 8 Gy) with or without medium exchange 10 min or 24 h after irradiation respectively. Sodium selenite was dissolved in sodium chloride or aqua bidest. Colonies were fixed with 70% (v/v) ethanol and stained with crystal violet after irradiation and counted manual by scoring only colonies with a minimum of 50 cells by phase contrast microscopy (Nikon Eclipse TE300, Tokyo, Japan). Clonogenic assays were carried out in three independent experiments performed as duplicates for each experimental approach.

### Metabolic activity

The effect of sodium selenite on cell metabolism of both cell lines was detected by cytotoxicity assay (EZ4U; BIOZOL Diagnostica GmbH, Eching, Germany) in 96-well plates at a density of 1 × 10^4^ cells per well in six replicates for each experimental approaches, which were carried out in three independent experiments. Sodium selenite was added at concentrations of 0 µM to 100 µM to the cells 24 h after seeding and 10 min before irradiation (0 Gy or 8 Gy). After a 24 h treatment period of cells with sodium selenite metabolic activity was measured photometrically after a 2 h (A549 cells) or 4 h (BEAS-2B cells) tetrazolium salt incubation.

### Cell cycle analysis

Confluent cells were seeded in an appropriate density followed by addition of sodium selenite 24 h after cell seeding. The irradiation with single-doses of 8 Gy or 0 Gy (control) as duplicates for each experimental approach was performed 24 h after sodium selenite addition and carried out in three independent experiments. 24 h or 48 h after irradiation cells were fixed and permeabilized 10 min in ethanol (70% (v/v), − 20 °C), and stained with propidium iodide (75 µM). Samples were measured on flow cytometer Cytomics FC 500 (Beckman Coulter, Krefeld, Germany). Analysis was performed using *Multicycle for Windows*, version 3.0 (Phoenix Flow Systems, San Diego, USA).

### Statistical analysis

Calculations were performed on the mean of at least three independent experiments. Statistical analyses were carried out using Student’s *t*-test. *P* ≤ 0.05 was considered as statistically significant difference.

## Results

### Inhibition of cell growth

**Fig. 1:**
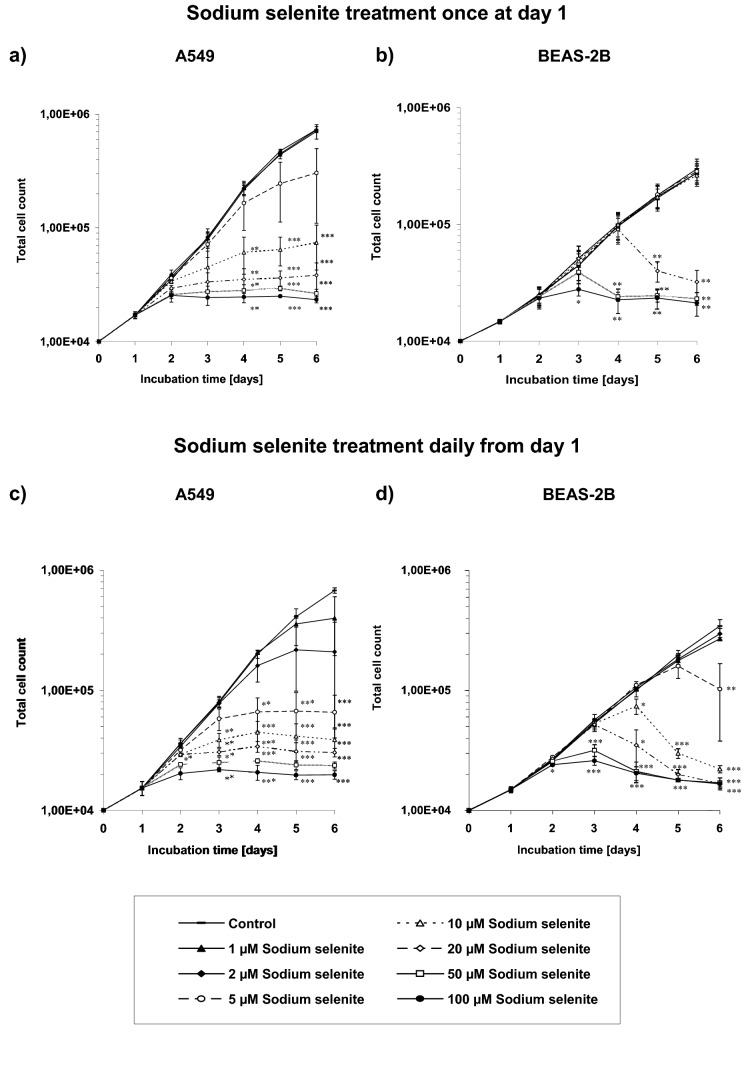
Growth curves of sodium selenite treated cells. The cells were seeded at day 0. Sodium selenite was added to the cells at day 1 of incubation after a 24 h cell adhesion period. Sodium selenite treatment was given once without medium exchange to **a** A549 cells, **b** BEAS-2B cells, or added daily without medium exchange to **c** A549 cells, **d** BEAS-2B. The solvent (sodium chloride) did not have any significant effect on the cell growth (data not shown). Error bars represent the standard deviation of three separate experiments; wells were assayed in triplicates in each of the different experiments. Significance was calculated for each day’s approaches (control versus treated sample). Asterisks illustrate significances *P ≤ 0.05, **P ≤ 0.01, ***P ≤ 0.001

The growth of cancer cells as well as non-cancerous cells was inhibited by sodium selenite, dependent on the concentration and incubation time of the substance. For singular sodium selenite treatment in A549 cells an inhibition of cell growth initially was determined at a low concentration of 5 µM sodium selenite at day 3 (48 h after non-recurrent sodium selenite application) and higher concentration of sodium selenite 24 h after application (Fig. 1a). In contrast the inhibitory effect of one-time treatment with sodium selenite on the growth of normal BEAS-2B cells did not start till concentrations of 20 µM sodium selenite and more (Fig. 1b).

For daily treatment of sodium selenite it could be observed that the growth of cancer cells was inhibited earlier and by lower concentrations of sodium selenite than by one-time treatment (Fig. 1c). Also, the growth of normal cells was already inhibited at a lower concentration (5 µM Na_2_SeO_3_) in comparison with a single application (20 µM Na_2_SeO_3_; Fig. 1d).

### Clonogenic survival

**Fig. 2:**
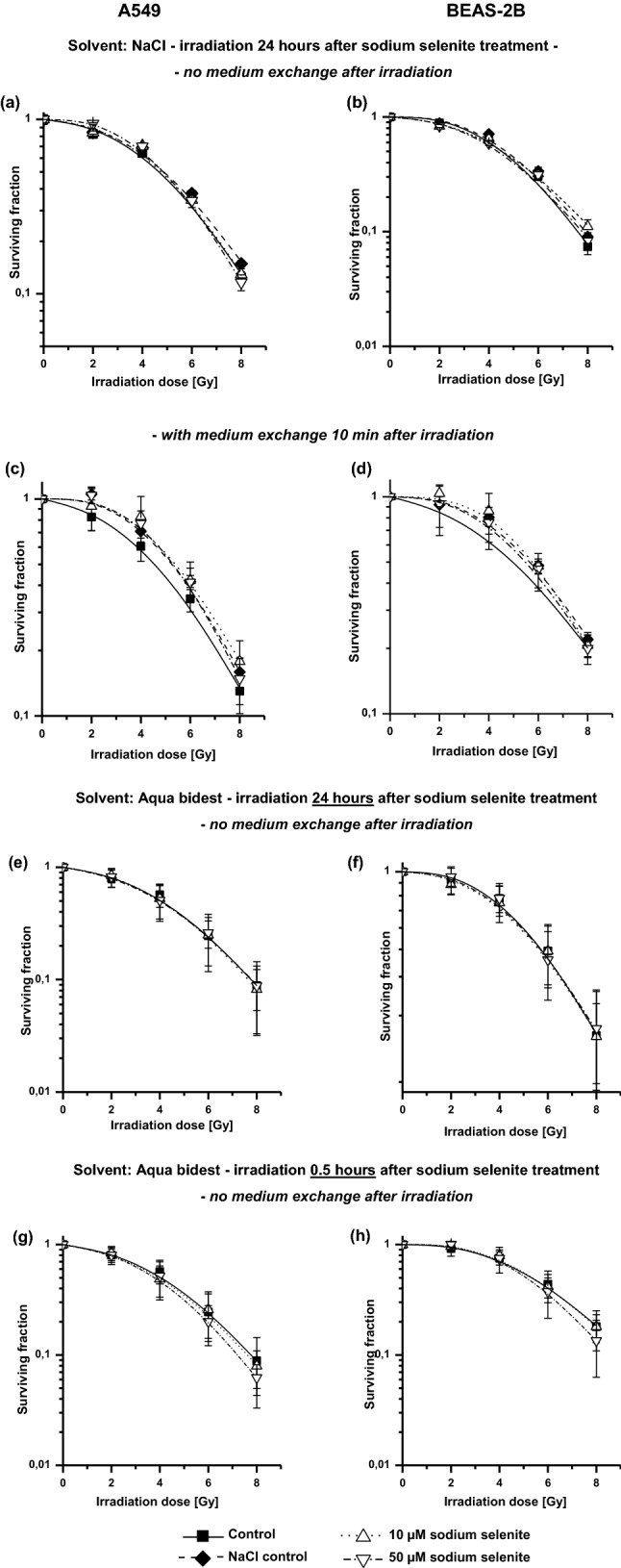
Clonogenic survival curves of sodium selenite treated A549 cells (**a**, **c**, **e**, **g**) and BEAS-2B cells (**b**, **d**, **f**, **h**) in combination with ionizing irradiation (2–8 Gy) or non-irradiation (0 Gy). Sodium selenite was added to the cells 0.5 h or 24 h before irradiation. Medium was exchanged 10 min after irradiation (**c, d**) or the medium was not exchanged after irradiation (**a**, **b**, **e**–**h**). Colonies were stained with crystal violet and counted manually by scoring only colonies with a minimum of 50 cells. Sodium selenite was dissolved in sodium chloride (**a**–**d**) or aqua bidest (**e**–**h**). The surviving fractions of treated cells were normalized to the plating efficiency of untreated controls (0 µM Na_2_SeO_3_; 0 Gy). Error bars represent the standard deviation of three separate experiments

The clonogenic assay was performed to determine the influence of irradiation in combination with sodium selenite on the clonogenic survival of both cell lines. Different treatment plans were tested, using different incubation times, and the solvents sodium chloride (NaCl) or aqua bidest. The survival fractions were shown as a function of radiation doses (Fig. 2).

In general, the survival of both cell lines decreased in accordance with the increased radiation dose in all experiments (Fig. 2a–h). It could be observed that the normal cells (Fig. 2b, d, f, h) showed similar radiation responses like the cancer cells (Fig. 2a, c, e, g), independent of the different treatment schedules with irradiation and sodium selenite. But the different treatment schedules as well as the choice of solvent (NaCl or Aqua bidest) clearly influenced the cellular radiation response.

When the cells were pre-treated for 24 h with sodium selenite (dissolved in NaCl) and no change of medium after irradiation was performed, a very slight protective effect was initially indicated for both cell lines (Fig. 2a, b). This effect markedly increased when the medium was changed 10 min after irradiation (Fig. 2c, d). A comparison with the respective curves of the solvent control mixtures (NaCl) revealed, however, that the initially suspected protective effect was caused by the influence of the solvent NaCl. For even the survival of the cells, which were treated only with the solvent NaCl, were well above the control curves of the untreated cells.

Subsequently, it was tested whether the synergistic effect of sodium selenite on tumour cells described in the literature may be caused by the use of another solvent. In a further experiment, therefore, the cells were treated with sodium selenite, which had previously been dissolved in double-distilled water instead of NaCl. But combined treatment with sodium selenite dissolved in aqua bidest did not affect the clonogenic survival of the cells, either on A549 cells (Fig. 2e) or on BEAS 2B cells (Fig. 2f) under this conditions.

In a further experimental approach, it was tested whether the modification of the length of pretreatment of cells with sodium selenite (dissolved in water again) influences clonogenic survival (Fig. 2g, h). So the drug administration was not 24 h but just before the irradiation (30 min). While 10 μM of sodium selenite had no influence on the radiation response of the cells, a slight radiosensitizing effect was observed in both cell lines after treatment with 50 μM sodium selenite 30 min before irradiation.

### Metabolic activity

**Fig. 3:**
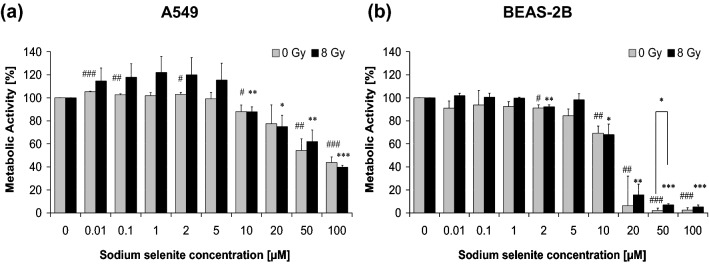
Metabolic activity of **a** A549 and **b** BEAS-2B cells treated with sodium selenite in combination with ionizing radiation (8 Gy) and non-irradiated controls (0 Gy). Sodium selenite was added to the cells 24 h after seeding and 10 min before irradiation. The metabolic activities of treated cells were normalized to the efficiency of untreated controls (0 µM Na_2_SeO_3_, 0 Gy; 100%). Error bars represent the standard deviation of three separate experiments; wells were assayed in six replicates in each of the different experiments. Significance was calculated for non-irradiated experiments related to non-irradiated (#) control (0 µM Na_2_SeO_3_; 0 Gy). Irradiated experiments were related to irradiated (*) control (0 µM Na_2_SeO_3_; 8 Gy). Asterisks/hash signs illustrate significances *^/#^*P* ≤ 0.05, **^/##^*P* ≤ 0.01,***^/###^*P* ≤ 0.001

The EZ4U assay was carried out to investigate the effect of sodium selenite on the metabolic activity of the cells (Fig. 3a, b). In A549 cells, after treatment with small doses of sodium selenite (0.01 µM to 5 µM), metabolic activity was unaffected in non-irradiated cells or increased in irradiated cells (8 Gy) respectively. From doses of 10 µM of sodium selenite or higher, metabolic activity decreased slowly but significantly steadily, independent of radiation exposure (Fig. 3a). For non-irradiated BEAS-2B cells, metabolic activity decreased slowly at a dose of 10 µM (69%), followed by a dramatic reduction at concentrations of 20 µM (6%) and more (Fig. 3b). The same strong influence of sodium selenite was observed by irradiated BEAS-2B cells compared to A549 cells. Generally, all sodium selenite-treated cells showed a slightly higher metabolic activity with irradiation compared to non-irradiation. The effect was significant in normal cells after treatment with 50 µM sodium selenite.

### Analysis of cell cycle

To investigate the cell cycle distribution of (a) A549 and (b) BEAS-2B cells, DNA histograms were generated by using flow cytometry. Histograms were used to determine the ratio of cell cycle phases. The analysis of the cell cycle distribution of untreated cells is shown in Supplements Fig. 1.

#### Cell cycle analysis 24 h after sodium selenite treatment (10 min after irradiation)

**Fig. 4:**
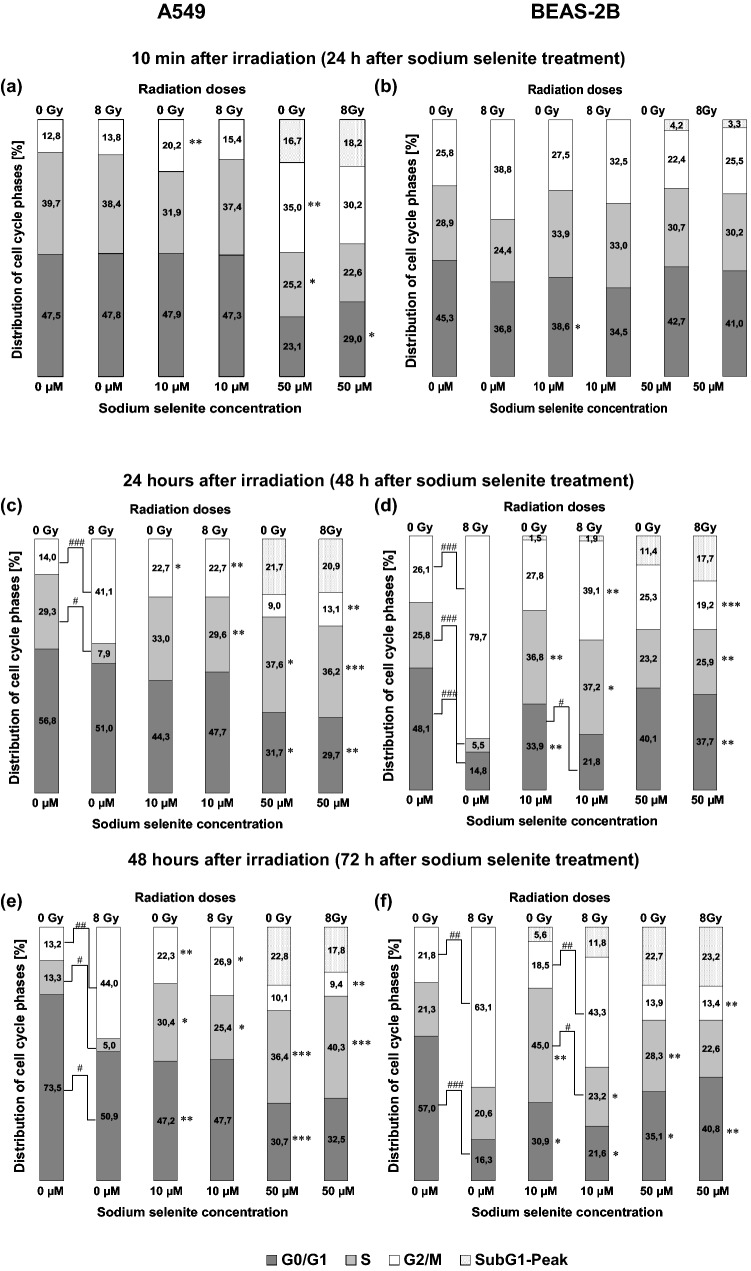
Cell cycle analyses of sodium selenite treated A549 (**a, c, e**) and BEAS-2B (**b, d, f**) cells in combination with ionizing radiation (8 Gy) or non-irradiation (0 Gy). Sodium selenite was added to the cells 24 h before irradiation. Cells were fixed 10 min (**a, b**), 24 h (**c, d**) or 48 h (**e, f**) after irradiation. For the three independent experiments significances were calculated in relation to controls without sodium selenite treatment (for non-irradiated experiments: 0 µM sodium selenite; 0 Gy; for irradiated experiments: 0 µM sodium selenite; 8 Gy) and illustrated by asterisks (*P ≤ 0.05, **P ≤ 0.005, ***P ≤ 0.0005). Hash signs illustrate significance between data from treatment with the same sodium selenite concentration, among non-irradiated (0 Gy) and irradiated (8 Gy) attempts (^#^*P* ≤ 0.05, ^##^*P* ≤ 0.005, ^###^*P* ≤ 0.0005)

Treatment with sodium selenite strongly influenced the distribution of cells in the cell cycle phases of both cell lines but in a different way. A 24-h sodium selenite treatment (Fig. 4a) in A549 cells with 50 µM had a strong influence on cell cycle distribution, resulting in a significant increase of cells in G2/M phase and a decrease of cells in S and G0/G1 phases. Additionally, a clear sub-G1 fraction could be detected at this high concentration of sodium selenite. In contrast, no great difference between 10 µM sodium selenite and untreated controls (0 µM sodium selenite) could be detected for the cancer cells. No difference between irradiation and non-irradiation were observed.

The normal BEAS-2B cells treated with sodium selenite (10 µM or 50 µM) showed a similar trend for cell distribution like cancer cells, with most cells being in phase G0/G1 followed by cells being in S and G2/M phases (Fig. 4b). But influence of sodium selenite was lesser than in tumour cells. A sub-G1 cell fraction could initially be detected at a concentration of 50 µM sodium selenite for non-irradiated and irradiated cells (Fig. 4b). Additional irradiation (8 Gy) had an effect on cell cycle distribution, resulting in an accumulation of cells being in G2/M phase using 0 µM and 10 µM sodium selenite. After treatment with an increased sodium selenite concentration (50 µM), no difference between non-irradiated and irradiated cells was observed, and the G2/M phase accumulation was abolished.

#### Cell cycle analysis 24 h after irradiation

Twenty-four hours after irradiation (Fig. 4c, d) for both cell lines without sodium selenite treatment a distinct arrest of cells in G2/M phase—which was more pronounced in normal cells (79.7%) than in cancer cells (41.1%)—could be observed. The additional treatment of irradiated cancer cells with 10 µM sodium selenite and 8 Gy did not influence cell cycle distribution compared to non-irradiated cells being treated with 10 µM (Fig. 4c). The percentage of normal cells being in G2/M phase increased from 27.8 to 39.1% (10 µM sodium selenite, 0 Gy or 8 Gy respectively) as a consequence of combined treatment. For both cell lines, a concentration of 50 µM sodium selenite resulted in a delay of cells into S phase and sub-G1 peak. However, clearly more BEAS-2B cells were accumulated in G2/M phase compared to A549 cells.

#### Cell cycle analysis 48 h after irradiation

The extension of incubation time to 48 h after irradiation (72 h after sodium selenite addition) showed similar effects on cell cycle distribution for A549 cells (Fig. 4e) as already observed in the analysis 24 h after irradiation (Fig. 4c). For BEAS-2B cells (Fig. 4f), treated with 50 µM sodium selenite cell fraction being in G2/M phase, decreased compared to an incubation time of 24 h after irradiation.

## Discussion

The main objective of this study was to compare the effect of sodium selenite on tumour and normal cells of the human lung under special attention of their influence on cellular radiosensitivity. In our study, it was shown that sodium selenite has an inhibitory effect on cell growth of both cancer (A549) and normal (BEAS-2B) cells. Sodium selenite-mediated inhibition of cell growth was already described for a lot of human cell types, from glioblastoma [25]), colorectal cancer [26], leukaemia [27], liver cancer [28], breast cancer [29], malignant mesothelioma [30], ovarian tumour [31], as well as lung adenocarcinoma [32, 33]. Several workgroups found out that sodium selenite can induce cell death by apoptosis [25, 26, 30]. In A549 cells, programmed cell death induced by sodium selenite was determined as well [32].

The metabolic pathway of selenium compounds is very complex. A significant step in the metabolism of selenite is the reduction of the drug, facilitating the incorporation into selenoproteins [34]. After supplementation with selenite an increase in the expression and activity of selenoproteins has been shown. Selenoproteins were described to influence the DNA damage repair by involving the redox regulation of signalling pathways and redox-sensitive proteins [35]. In addition, the activity of glutathione peroxidases is induced by selenite, which has also been described for lung cancer cells [36].

As mentioned in the introductory section, selenite can have antioxidant and prooxidative properties. The main mechanism of selenite cytotoxicity is its pro-oxidative property, where oxidative stress is caused by the generation of reactive oxygen species (ROS) and redox active metabolites. It was demonstrated that the drug is reduced by thioredoxin reductase (TrxR), the thioredoxin (Trx) and glutaredoxin (Grx) systems under formation of hydrogen selenide ion (HSe^−^) and redox cycling with oxygen. During this process ROS are generated [7, 34]. It has also been shown that reduction of selenite produces selenodiglutathione (GSSeSG), which is reduced to glutathione selenenylsulfide (GSSeH) by NADPH and glutathione reductase. Finally also HSe^−^ is formed. It is assumed that hydrogen selenide [H_2_Se; at physiological pH: hydrogen selenide ion (HSe^−^)] is a common metabolite at which all selenium metabolic pathways cross [7]. It is referred to as the "selenide pool". But due to the high reactivity with O_2_ and metals, it cannot be assumed that HSe^−^ freely exists in large concentrations.

The selenite-mediated ROS generation (e.g. the generation of superoxide, hydrogen peroxide, hydroxyl radicals) by the drug itself and its metabolites was observed in several studies and has been associated with oxidative stress leading to DNA strand breaks and apoptosis in various cancer cell types [37–39]. In addition, it has been described for a number of cancer cell lines that selenite induced mitochondria mediated apoptosis. Selenite leads to a decrease in the mitochondrial membrane potential and to the release of cytochrome c into the cytosol, which ultimately activates the apoptotic pathway [7, 40]. Furthermore selenite is able to oxidize protein thiols, not only via ROS generation but also directly. This leads to a selenite mediated cross-linking of mitochondrial proteins inducing permeability transition of mitochondria and cell death [41]. It could be shown that sodium selenite leads to excess production of ROS in A549 cells, which causes autophagy and cell death [33, 42]. Furthermore, analyses of metabolomics and gene expression showed that sodium selenite disturbs glycolysis, blocked the citric acid cycle, and polyamine metabolism, suppressing glutaminase 1 (GLS1) expression in A549 cells [31].

In our study, the growth of A549 tumour cells was more sensitive to sodium selenite than the normal BEAS-2B cells. To achieve the same inhibitory effect on cell growth of normal cells, treatment time or concentration of sodium selenite had to be increased in comparison with the cancer cells. In several studies, a higher cytotoxicity of selenite towards tumour cells compared to normal cells at a comparable dose was reported as well [29, 30, 32, 43]. In glioma cells, sodium selenite was described as being about two-fold more cytotoxic than in normal astrocytes [25]. It was also shown that prostate cancer cells of three patient-matched pairs [44], as well as PC-3 cells and DU145 cells [45] were more sensitive to treatment with sodium selenite than normal prostate cells.

There is evidence that the level of intracellular sulfhydryl (SH) compounds seems to be an important factor for the growth inhibition effect of sodium selenite. Studies of [46] showed that A549 cells, having high levels of SH compounds, are more sensitive to sodium selenite treatment than normal lung fibroblast cells with lower levels of SH compounds. As a mechanism of tumour-selective cytotoxicity, other groups assume the importance of extracellular thiols for the uptake of selenium from selenite [47].

To assess the influence of sodium selenite on the cellular radiation response, the clonogenic survival of cells was determined using clinically relevant doses from 2 to 8 Gy. Various reports assumed that treatment with sodium selenite during radiotherapy may have a greater impact on tumour cells whereas normal cells are more likely to be protected from radiation [7]. This was attributed—amongst others—to the higher sensitivity of the tumour cells to oxidative stress mediated by sodium selenite. However, in our study, no general difference in radiosensitivity between the tumour (A549) and normal (BEAS-2B) cells under the influence of sodium selenite could be observed. But we could demonstrate on both the tumour as well as the normal cell line that the cell response to irradiation of sodium selenite treated cells depends very strongly on the chosen experimental conditions, such as the chosen solvent of sodium selenite (NaCl or H_2_O), and the performance or lack of media exchange after irradiation. As far as the effect of the medium change is concerned, the following assumption can be made. As already described, the cytotoxic effect of selenite is based on its pro-oxidative properties and thus on the formation of ROS and redox-active metabolites. Ionizing radiation also induces the formation of ROS, mainly from the radiolysis of water. The majority of all reactive intermediates formed (e.g. radicals) should also be in the surrounding medium after the cells have been treated. These could be eliminated by changing the medium. Since the half-life of the reactive intermediates is only very short and the medium change did not take place until 10 min after the irradiation, it could be assumed that these were already reduced and the effect of the medium change is not really strong. However, the comparison of the survival curves with and without a change of medium showed a difference, which is certainly due to the elimination of the reactive intermediates. An initially suspected protective effect (24 h pre-treatment with sodium selenite dissolved in NaCl) turned out on closer inspection to be the effect caused by the influence of the solvent NaCl. In contrast to this, a slight radiosensitizing effect was exhibited in both cell lines when sodium selenite (50 μM) was dissolved in H_2_O, pretreated 0.5 h before irradiation. Using deviant experimental conditions (I: solvent H_2_O, pretreatment 24 h, no medium exchange; II: solvent NaCl, pretreatment 24 h, no medium exchange), no effect on radiation response could be determined. These different results that were observed, caused by the chosen experimental conditions, may explain the contradictory effects of sodium selenite on the cellular radiation sensitivity described in the literature. There are reports for sodium selenite from radiosensitizing [13, 14] to radioprotection [15] to no influence on radiation response [16, 17]. On the basis of our results, could not confirm the hypothesis of Schueller and co-worker that sodium selenite in low concentrations (< 5 µM) is radioprotective, while high doses cause radiosensitization [13].

On the basis of the results from the dose–effect curves in the clonogenic assay, an example dose of 8 Gy was used for the radiation exposure of the cells for further studies.

To verify the inhibitory effect of sodium selenite on cellular growth and the influence on radiation response, metabolic activity of non-irradiated as well as irradiated cells was investigated. From doses of 10 µM sodium selenite or higher, metabolic activity of both cell lines decreased; from 20 µM and up, normal cells significantly stronger than that of cancer cells. Similar results were described in the literature. For human osteosarcoma U2OS cells until 10 µM sodium selenite, no effect was seen using MTT assay [48]. Also, as in our study, in human colon cancer cells HCT-116 sodium selenite concentrations of 10 µM and higher significant reductions of metabolic activity were seen [3]. Generally, the amount of formazan product is proportional to the number of metabolically active viable cells. For a long time, it was assumed that the conversion of tetrazolium salts occurs exclusively through mitochondrial succinate dehydrogenases. In the meantime, it is known that tetrazolium salts can be converted by multiple oxidoreductases located both inside and outside the mitochondria. After the influence of sodium selenite, however, significant inhibitory effects on cellular mitochondria have been described. It is known that sodium selenite generates oxidative stress with ROS formation which inhibit the mitochondrial membrane potential of cells [42]. Studies verified—via measurement of mitochondrial membrane potential and superoxide anion—that sodium selenite induced damage of mitochondria in human malignant glioma cells U87MG, T98G, A172, U343, and U251, but not in human astrocytes from primary cultures [39]. Superoxide anion production and a decrease of mitochondrial membrane potential by sodium selenite in transduced human prostate cancer cells (LNCaP) were discussed as possible reasons for the damage of mitochondria cells [49].

The effect of combined treatment with sodium selenite and irradiation on cell cycle was tested in both cell lines via flow cytometry. After irradiation alone, both cell types showed the for irradiation known typical G2/M arrest. This effect was more pronounced in normal cells than in tumour cells. Under treatment with sodium selenite, the G2/M arrest caused before by radiation exposure was abolished (especially at 50 μM), 24 h and 48 h after irradiation. Also, in LNCaP and PC3 prostate cancer cells, no G2/M arrest could be found 24 h after sodium selenite treatment [50]. We have observed that while the tumour cells increased in the S phase, the normal cells resulted in an increase in G0/G1 phase in comparison with only irradiated cells. For DU145 human prostate tumour cells, Jiang and co-worker also saw an S phase arrest after sodium selenite, while they observed no elevation of cells in G2/M phase [51]. In our study, after higher doses (50 µM) of sodium selenite, both cell lines showed more cells in sub-G1 phase. Sub-G1 cells indicates apoptosis. Cell death via apoptosis is characterized by DNA fragmentation. On the basis of their reduced DNA content, including nuclear condensation, which can be detected by flow cytometry as sub-G1 peak, apoptotic cells can be identified and quantified [52]. The results in our study revealed that the percentage of sub-G1 cells increased after application of sodium selenite. Therefore, sodium selenite treatment may led to a potent increase in apoptotic cells in a dose-dependent manner. Our results are in agreement with earlier findings, in which the induction of apoptosis in cells treated with sodium selenite was also detectable through the increase in the sub-G1 phase [42, 53]. For example, an increase in cells in the sub-G1 phase under the influence of sodium selenite was also observed in HCT-116 human colon cancer cells [3].

The biological activity of selenite depends on the activity of the various metabolic pathways and the redox status of the cells/tissues [7]. Of course, it must be noted that the results of the in vitro studies cannot be directly transferred to the in vivo situation. Due to the different intracellular redox environments and the different available metabolic pathways, the selenite metabolites already differ in vitro and in vivo.

In summary, in the present work, no general difference in radiosensitivity between the investigated tumour (A549) and normal (BEAS-2B) cells under influence of sodium selenite was observed. Our results show that sodium selenite can mediate different effects on radiosensitivity of the same cells: from an initially suspected but ultimately no real radioprotection to no effect on radiation response up to radiosensitizing, dependent on the chosen treatment conditions like the solvent of sodium selenite and incubation time media exchange after irradiation. These results support the previous results on the potential for the use of sodium selenite in radiation therapy, but also illustrate the urgent need and importance of further elucidating the mechanisms of action of sodium selenite with special attention to the experimental conditions.

## Electronic supplementary material

Below is the link to the electronic supplementary material.

Supplements Fig. 1 Analysis of the cell cycle distribution of a) A549 and b) BEAS-2Bcells using flow cytometry. The DNA histograms of the cell cycle show the distributionof untreated cells (DOCX 2823 kb)

## Data Availability

Data supporting this study are provided in the results section or as supplementary information accompanying this paper. Further datasets used and/or analysed during the current study are available are stored by the authors at the University Medical Center Rostock.

## Abbreviations

BrdU: Bromodeoxyuridine
DMEM: Dulbecco’s Modified Eagle’s Medium
FBS: Foetal bovine serum
NaCl: Sodium chloride
PBS: Phosphate buffered saline
