# Supplementary material for: The solvent and treatment regimen of sodium selenite cause its effects to vary on the radiation response of human bronchial cells from tumour and normal tissues
Source: Med Oncol. 2020 Nov 18;37(12):115. doi: 10.1007/s12032-020-01437-y (PMC7671986; doi:10.1007/s12032-020-01437-y)
Supplement: Supplementary file 1 — Supplements Fig. 1 Analysis of the cell cycle distribution of a) A549 and b) BEAS-2Bcells using flow cytometry. The DNA histograms of the cell cycle show the distributionof untreated cells (DOCX 2823 kb) [file 12032_2020_1437_MOESM1_ESM.docx]

**Supplements Fig. 1**

| **a) A549** | **b) BEAS-2B** |
| --- | --- |
| **b.)**  **a.)** | |
